# Quality of Life analysis of patients in chronic use of oral anticoagulant: an observational study

**DOI:** 10.1186/1477-7525-9-91

**Published:** 2011-10-25

**Authors:** Geisa de Queiroz Almeida, Lúcia de ACB Noblat, Luiz Carlos Santana Passos, Harrison Floriano do Nascimento

**Affiliations:** 1 Medicine and Health Graduation Program, Federal University of Bahia, Rua Odilon Dorea n°1 edf. Suzana apt° 202 Brotas, Salvador, Bahia, 40.285-450, Brazil; 2 Departament of Medicines, Pharmacy School Federal University of Bahia, Rua Barão de Geremoabo S/n Campos Universtitário de Ondina, Salvador, Bahia, 40.170-115, Brazil; 3 Departament of Medicines, Medical School Federal University of Bahia, Rua Padre Feijó n° 240 Ambulatório Magalhães Neto 3° andar, Salvador, Bahia, 40.130-170, Brazil; 4 Health Economy Departament of Professor Edgard Santos University Hospital, Federal University of Bahia, Rua Augusto Viana s/n, 2° andar, Salvador, Bahia, 40.110-060, Brazil

## Abstract

**Background:**

Treatment with oral anticoagulant may influence the quality of life perception as it promotes changes in the patient's life, not offering an evident symptomatic relief and presenting well defined risks, such as bleeding. In this trial, the influence of chronic use of anticoagulants on the quality of life perception has been analyzed in patients assisted at the anticoagulation outpatient unit.

**Methods:**

The health related quality of life was evaluated through a cross-section study with a sample composed of 72 patients seen from July 23, 2009 to September 2, 2010 at the Anticoagulation Outpatient Unit of the Federal University of Bahia's University Hospital. The study's population was composed by patients with atrial fibrillation and mechanical heart valve. The patients were submitted to two quality of life evaluation questionnaires: a generic questionnaire - the Medical Outcomes Study SF-36 Health Survey (SF36) - and a specific questionnaire - the Duke Anticoagulation Satisfaction Scale (DASS).

**Results:**

The quality of life perception of the patients studied, based on both the DASS and the SF36, was positive regarding the treatment with oral anticoagulant. The SF36 presented an average score of 62.2 (± 20.0). Among the SF36 evaluated domains, the physical-emotional aspect was the most compromised one regarding life quality perception. The DASS presented an average score of 67.1 (± 18.2) and the domain presenting a greater compromise was the one related to the treatment inconveniences (annoyances, burdens and obligations). Previous hemorrhagic event, comorbidities, drug interactions with medicines that increase the anticoagulant effect, lower education level in the SF36 and younger age group influence a more negative perception of the quality of life, whereas lower education level in the DASS and the duration of treatment for more than 1 year offer a more positive perception.

**Conclusion:**

Patients seen at the anticoagulation outpatient unit of the University Hospital of Federal University of Bahia/Brazil had a positive perception of the treatment. Factors such as hemorrhagic event, comorbidities, drug interactions, education level, age group and duration of treatment have an influence on the quality of life perception.

## Background

The quality of life (QoL), according to World Health Organization - WHO (1947), is a wide concept involving several factors affecting the life of a person, such as social condition, health, economical status, satisfaction and welfare. The QoL aims at getting to know the difficulties of living with a disease, and such difficulties may be subjective and diverse for each patient [1]. Therefore, the QoL is related to the perception the subject has of his/her health status and of the disease effects on his/her life, involving both physical and functional aspects besides emotional and social ones.

Long-term oral anticoagulation is a required treatment for specific patients with atrial fibrillation (AF) and mechanical heart valve (MHV) and for the prophylaxis of strokes and other thromboembolic events. However, a therapy with coumarinic agents (warfarin and phenprocoumon) is complicated due to the variability of the biological effect, its narrow therapeutic index and potential occurrence of thrombotic or bleeding events.

The oral anticoagulant therapy control is complex and requires frequent analytical checking, with several visits to health units to control the INR (International Normalized Rate). The chronic use of oral anticoagulants can promote an influence on the patient's perception regarding his/her QoL and health status due to the changes it promotes in the patient's life style, and mainly because patients are submitted to a treatment that brings no symptomatic benefit, but presents a well-defined risk. Thus, it is noted the importance of getting to know the difficulties involving living with oral anticoagulant treatments.

Few previous studies have evaluated QoL in patients presenting cardiopathy and chronic use of oral anticoagulant drugs. Casais[2], in a study conducted in Buenos Aires, Argentina, has shown a positive perception of to the use of oral anticoagulant drugs among patients presenting all the indications. Patients with cardiopathies were the majority of the studied population.

The objective of the study was to analyze the QoL of patients with chronic use of oral anticoagulants in a university hospital in the state of Bahia through the obtained scores via the application of the QoL SF36 and DASS instruments, besides evaluating the influence of clinical and demographic factors in the perception of quality of life. This regional evaluation is important, considering that cultural and subjective aspects play an important role in the negative or positive perception of patients submitted to anticoagulation treatment.

## Method

QoL was evaluated through a cross-section study with a sample composed of 72 patients seen from July 23, 2009 to September 2, 2010 at the Anticoagulation Outpatient Unit of the University Hospital of Federal University of Bahia.

The sample was composed of patients with AF and MHV, regardless gender or race, living in Salvador - the capital city - and in the countryside part of Bahia, under chronic treatment with oral anticoagulants. The inclusion criteria were: patients with AF and/or MHV, under chronic treatment with oral anticoagulant, who agreed to sign an informed consent form. The exclusion criteria were: anticoagulated patients by means other than AF or MHV and who were not capable of answering all the questions in the QoL questionnaire applied.

The sampling type was chosen according to its convenience. The patients were identified through the cardiology service the anticoagulation outpatient, and those who fulfilled the inclusion criteria were invited to take part in the research. The invitation and questionnaire application both took place while patients waited between the execution of the PT and INR exam and the result for their appraisal.

The anticoagulation outpatient department of Professor Edgard Santos university hospital complex was chosen as site of this research since it is a public service of high patient demand for PT and INR exams, and because it offers specialized assistance to such patients, with a team composed of a cardiologist doctor and a pharmacist. The service consists of an evaluation of the anticoagulation level of the patient (PT and INR) considering the reason for using the anticoagulant and risk factors, the evaluation of the interactions of the anticoagulant and others medicaments and/or food, the adjustment of the oral anticoagulant's dose when necessary, the orientation of the patient regarding the used dose, the existing interactions, use time, the importance of his/her adherence to the treatment and the evaluation of hemorrhage signals when INR is elevated.

Data collection was conducted through the application of two questionnaires, one generic and one specific, to evaluate the subject's QoL. The QoL generic instrument was the SF36, which involves aspects such as physical function, performance status, pain, overall health status, mental health, emotional and social aspects, and vitality. It is a multidimensional questionnaire and its scores vary from 0 to 100, with zero regarding the worse and 100 the best QoL for each domain. The specific instrument DASS is an specific scale to evaluate the QoL of patients under treatment with oral anticoagulants, involving limitations, displeasure and burden (daily displeasure, such as regular return for medical visits and wait for blood tests results), and positive psychological impacts. The items presenting the lowest scores show the higher satisfaction with QoL[3]. The overall score varies from 25 to 175. The instrument is divided in three domains: limitation (score from 9 to 63), treatment inconvenience (score from 8 to 56) and psychological impact (score from 8 to 56) [4]. There was no adaptation of the questionnaire for use with the studied population. An analysis of the subscales was made by adding the answers to the items (varying from 1 to 7 for each item) in each domain and reaching the total score by adding up all the questionnaire items. Clinical, demographic, and socioeconomic variables were collected by a questionnaire formulated by the investigator to obtain such data, which was applied at the same moment of the generic and the specific questionnaire.

The QoL questionnairs (both generic and specific) were applied via a paper form by a pharmaceutical professional. None of the questions in the QoL questionnaires was omitted or altered.

The descriptive data analysis was made by using the frequencies for the categorical variables and the continuous quantitative variables were described based on their mean and standard deviation. Database typing and analysis were made at Excel 1.0 and SPSS 17.0 softwares.

This study was submitted and approved by the Research Ethics Committee of Climério de Oliveira Maternity Hospital (OPINION/ADDITIVE RESOLUTION No. 153/2009), and all patients included in the study sample have signed the informed consent form, according to the directives of Resolution 196/96 of the National Health Council.

## Results

Table 1 shows the subjects characteristics. The study's population was composed of 72 patients of both genders. Patients under 18 were not included. The number of patients inquired for consent in order to take part in the research was 73, and all 73 consented although 1 was excluded for not being capable of answering the questionnaire.

**Table 1:** Study population characteristics

Age range (years)	22 to 87
Female (%)	62.5

Race (%)	
Black	48.6
White	20,8
Mulatto	9,7
Other	20,8

Education (%)	
Illiterate	9.7
Incomplete primary education	45.8
Complete primary education	6.9
Incomplete high school	18.1
Complete high school	18.1
Incomplete University course	1.4

Married (%)	52.8

Anticoagulation reason (%)	
AF	48.6
MHV	45.8
AF and MHV	5.6

Existence of comorbidities (%)	59.7

Use of drugs in addition to an anticoagulant (%)	98.6
Increase the anticoagulant effect	45.8
Decrease the anticoagulant effect	2.8

Anticoagulant (%)	
Warfarin	72.2
Phenprocoumon	27.8

Treatment length (%)	
Less than 01 year	16.7
Between 01 to 08 years	52.8
More than 08 years	26.4

Existence of previous bleeding (%)	
Yes	37.5
No	62.5

Serious Hemorrhage Percentage and slight bleeding/bruise*	
Slight hemorrhage/bruise	92,3
Serious Hemorrhage	7,7

Hospitalization after treatment with anticoagulant due to complications with anticoagulant or for its dose adjustment. (%)**	
Yes	20.8
No	79.2

Required emergency care due to anticoagulation (%)**	
Yes	38.9
No	61.1

* Considering slight hemorrhages those whose conduct restricts to clinic observation or anticoagulant dose adjustment, and serious hemorrhages those which require anticoagulant suspension, blood replacement and/or internment.

** These answers are based on patient responses and not validated by review of the medical records

The DASS analysis showed a mean QoL score of 67.1 (± 18.2). The mean score of different domains composing the instrument is described in Table 2. Among the domains evaluated, the domain regarding treatment inconveniences (annoyance, burden, and obligations with treatment) showed a higher compromise of the QoL perception in the chronic treatment with an oral anticoagulant.

**Table 2:** Mean Score of the domains in the Specific Questionnaire DASS

	*Mean*	*Standard deviation*
Limitation with treatment	21.8	0.7

Inconvenient with treatment	23.7	2.0

Positive psychological impact	22.6	1.7

The SF36 analysis (Table 3) showed a mean score of 62.2 (± 20.0). The compromising of the different domains composing this instrument, in ascending order, were social aspects, overall health status, mental health, vitality, performance status, pain, emotional and physical aspects.

**Table 3:** Mean Score of the domains in the Quality of Life Scale SF36

	*Mean*	*Standard deviation*
Performance Status	60.1	25.7

Physical appearance	52.4	41.1

Pain	58.8	28.0

Overall health status	65.5	21.6

Vitality	60.3	22.1

Social aspects	80.4	23.8

Emotional aspect	57.9	42.6

Mental health	61.6	18.0

Several demographic and clinical aspects described in Table 4 may positively or negatively influence the QoL perception. The QoL perception among the MHV and FA patients may present a variation either in the SF36 or in the DASS. MHV patients presented a better QoL perception in comparison to FA patients.

**Table 4:** Factors that influence the perception of QoL

Factors that influence positively the perception of QoL	Factors that influence negatively the perception of QoL
Low education level (DASS)	Previous hemorrhagic event

Treatment length superior to 1 year	Presence of comorbidities 20,8

	Drug interactions

	Low education level (SF36)

	Younger age group

## Discussion

Most of the patients studied, based on the DASS and SF36, showed a positive perception regarding the QoL when in treatment with oral anticoagulant. Such finding in regard to the overall QoL is similar to that noted in the study of Casais [2], in which most patients showed a positive perception of the oral anticoagulant treatment.

The SF36 and the DASS proved to be adequate instruments to evaluate the patients' QoL. Both instruments are easy to use and self-explanatory. The specific DASS instrument offers points in the anticoagulant treatment that may influence the treatment directly or indirectly, such as fear of bleeding, changes of behavior, limitations towards treatment, etc. Applying the two questionnaires at the same time took quite a while, about 30 to 40 minutes for each patient, as the DASS questionnaire consists of 25 questions and the SF36 has 11 questions and 36 items.

The QoL perception of the study population showed a slight variance within the age group. Patients ranging from 41 to 65 years old showed a better QoL score when compared to younger patients and patients over 65 years old. Gadisseur [5] evaluated in his study the effect on the QoL of different modalities of treatment - self-handling and handling by specialized anticoagulation clinic. In this study [5], it has been observed that younger patients had better satisfaction with the anticoagulant treatment than older patients. Therefore, the variation in the QoL perception between young patients and older patients is in accordance with such study.

A higher tendency to the positive perception on QoL was observed in patients with lower education level in DASS, for which illiterate patients showed a more positive perception of QoL, while for SF36 a better perception of QoL is associated to a higher education level, except for incomplete college education. According to Casais [2], the positive perceptions more present in patients with low education level (illiterates or who only completed elementary school) may be related to the association of the anticoagulant treatment to sensations of health improvement.

According to Casais, the negative perceptions regarding oral anticoagulant treatment was more present in patients at the beginning of treatment, usually less than 1 year of treatment. This was also observed in this study, in the two QoL measurement instruments, in which patients with treatment duration below 1 year had a higher compromise regarding QoL. Patients between 1 and 8 years of treatment and patients with over 8 years of treatment showed, increasingly, a more positive perception of the treatment.

Regarding the existence of a bleeding event, patients who had already had previous bleeding, regardless the bleeding process severity, had a more negative perception of QoL, in both evaluation instruments. The domain showing higher compromise was the physical aspect in SF36 and annoyance and burden with treatment in DASS. Lancaster [6] noticed that patients who had a bleeding episode showed a significant decrease of health perception and concludes that, generally, the warfarin therapy is not associated with a significant QoL reduction, unless when a previous bleeding episode occurs. While Casais [2] showed that the negative perceptions related to anticoagulant treatment was more evident in patients worried about bleeding risks, but that bleeding episodes were not associated with negative QoL perceptions.

Variables such as drug interactions and comorbidities may also promote changes in QoL perception. Regarding the drug interaction variable, it was observed that patients using drugs that increase the effect of anticoagulant have higher QoL compromise when compared to patients who use drugs that reduce the anticoagulant effect.

The existence of comorbidities promotes a less positive perception of QoL compared to patients without comorbidities. This fact may be related to risk factors that are characterized, most of the time, by the presence of some comorbidities, as it occurs with FA patients, who present as risk factors for stroke, age >75 years, hypertension, thyrotoxicosis, diabetes, cardiovascular disease, congestive heart failure and stroke history, transient ischemic attack or thromboembolism [7].

According to Abhay [8], treatment complications with the oral anticoagulant, such as bleeding, and the inconvenience of required periodic monitoring, may reduce patients' QoL. However, patients may be more exposed to the potential consequences of not being treated with an anticoagulant, such as stroke, and less annoyed with the adverse effects of an oral anticoagulant. An observation study [9] showed that patients at high stroke risk valued more the fact of avoiding stroke than avoiding bleeding.

The QoL of patients with AF and MHV may present some variance on both research instruments. In the study, there was a positive trend of the QoL perception in patients with MHV in comparison to patients using an anticoagulant due to AF. In his study Casais [2] also observed a variation of the QoL scores depending on the indication for treatment with oral anticoagulant and showed that patients with AF feared more bleeding than patients with heart prosthesis, despite their having a lower absolute risk of bleeding [2]. The higher negative perception of patients with AF may be related to factors of the disease, in addition to the fact that, for patients with AF, the choice of anticoagulation is made according to the risk/benefit ration, and, therefore, it is limited to patients at moderate or high risk of stroke [7].

## Study Limitations

The current study was conducted within a specialized service to follow-up patients using oral anticoagulants that provides individual care, involving the control of anticoagulation and guidance on the treatment, drug and food interactions, and promotion of self-care, which might have lessened the treatment adverse aspects and, as a consequence, have contributed to a more positive attitude regarding the QoL. The DASS instrument used has been validated in Brazil, where the study was carried out, by Flavia Martinelle Pelegrino, having cultural, conceptual and semantic equivalence, thus not presenting any additional limitation to the work, besides its results matching the SF36's. The questionnaire SF36 was translated and validated in 1997 by Rozana Mesquita Ciconelli presenting cultural equivalence [10].

## Conclusion

This study showed that most patients under chronic treatment with oral anticoagulant showed a positive perception of QoL when attending an anticoagulation specialized outpatient service. However, this perception was influenced by demographic and clinical variables, which had a positive effect on the quality of life (low education level on DASS, duration of treatment superior to 1 year) or a negative one (previous hemorrhagic event, comorbidities, drug interactions with medicines that increase the anticoagulant effect, low education level on SF36 and younger age group). Since the QoL instrument was not applied at two different moments (before and after the treatment with oral anticoagulant) it can't be said that the anticoagulation increased or decreased the QoL perception of the patient, although the cross-section study permits to describe the QoL perception of the studied population. This study is an important contribution to the understanding of this subject, since most of the information available about the relationship between QoL and anticoagulation comes from researches that have been conducted in other countries, whose extrapolation for the Brazilian reality is limited by cultural and socio economic differences and by the way the health system is organized.

## List of abbreviation

(DASS): Duke Anticoagulation Satisfaction Scale; (SF-36): Short-Form 36 Generic Health-Related Quality of Life Scale; (PT): Prothrombin time; (INR): International Normalized Ratio; (QoL): Quality of Life; (WHO): World Health Organization;  (AF): Atrial fibrillation and (MHV): mechanical heart valve.

## Competing interests

The authors declare that they have no competing interests.

## Authors' contributions

GA contributed to study concept and design, data collections, statistical analysis, data interpretation, manuscript preparation, critical review of intellectual content. LN contributed to study concept and design, data interpretation, supplementary analyses, manuscript preparation and revising and critical revisions of the manuscript. LP contributed to conceptualization, revising the manuscript, supplementary analyses, and critical revisions of the manuscript. HF contributed to study concept and design, statistical analysis and data interpretation. All authors read and approved the final manuscript.
